# Hospital and Institutional News

**Published:** 1914-01-03

**Authors:** 


### HOSPITAL AND INSTITUTIONAL NEWS.

### STATE HOSPITALS WON'T BE TOLERATED BY ENGLISHMEN.

During 1913, at the meetings of the British Hospitals Association, of the governors of particular hospitals, at gatherings of the medical profession, in political circles, and at various assemblies, where people of the same views, or varied views, have met, there has been an increasing tendency to grow gloomy over the monetary prospects of our voluntary hospitals. The utterers of this loose talk have usually been men who pose as informed above their fellows. It therefore may be well to say plainly that loose discussions of State control or State hospitals are to be deprecated, because familiarity is apt to breed contempt. The nations of the world outside these islands afford startling proofs of the evils, and we may even say of the cruelties and hardships, which might rapidly attach to hospital treatment if this was to be confined exclusively to State hospitals. State hospitals have been tried under most favourable conditions in regard to unlimited supplies of money and brains and skill. But the State system has everywhere given so-called science an uncontrolled freedom to conduct treatment from a purely scientific point of view, with the result that the patients soon come to be regarded as mere material for science, and the humanity side of the hospital as we have it in these islands, which makes the comfort and well-being of the patients a paramount consideration, has necessarily to take a back seat. The working and industrial classes of these islands have had no experience of State hospitals such as those we have described, and they may thank God that they are free from the trials, discomforts, and hardships which sometimes attend residence in a hospital so constituted. That is why, from actual knowledge of the various systems of hospital administration that the world has evolved to the present day, we are convinced and unyielding supporters of the voluntary hospitals. These hospitals are the most humanising of medical institutions, for in them the patient is safeguarded and given all that makes life best worth living to a free people in a free country.

### A CENTRAL HOSPITAL BOARD FOR BRADFORD?

The enlargements and improvements to the Bradford Eye and Ear Hospital were inaugurated in time for the re-opening to precede the beginning of the New Year. The opening was made memorable by the suggestion of Mr. George Priestman, chairman of the house committee of Bradford Royal Infirmary, who advocated a common board on which all the charitable institutions in Bradford could be represented. He was sure that the Bradford Royal Infirmary would go a long way towards meeting such a suggestion. We shall be curious to see if this meets acceptance, for few things have been more curious than the hesitations and difficulties which at the start beset such schemes—witness the long-suggested co-ordination among the Birmingham and Midland Hospitals. On the other hand, the success which has attended such co-ordinations as the great funds, and particularly the King's Fund, has been phenomenal. Should Bradford follow suit?

### ANOTHER FORTUNE FOR THE HOSPITALS.

The announcement of the Wernher bequest of nearly half a million to King Edward's Hospital Fund for London is quickly followed by the news that through the death of Mr. Geoffrey Charles Ansell at the age of seventeen a sum of more than £250,000 becomes available for London hospitals and charities, of which £50,000 is earmarked for King Edward's Hospital Fund. The other hospital bequests are recorded in detail in another column: here we will only tell the story of the Ansell fortune, noting meanwhile that among other institutions receiving a similar sum Charing Cross Hospital is to receive £10,000, a gift as likely to be as acceptable as any in the list. The father of the boy, through whose death in a nursing home on December 21 this fortune becomes available for charity, was Mr. Charles Ansell, a member of the firm of stockbrokers, who died in 1905, leaving £305,951 net personalty. His will directed the payment of certain legacies, and subject to them that the estate should be put in trust for his son till he came of age. After providing for his education the boy was to receive £2,000 a year until he became twenty-five, after which the estate was to be held in trust for him for life, with remainder to his issue as he might appoint subject to any appointment for the life interest of a widow surviving him. It was also directed that in default of any issue to the son, and subject to the life interest of a surviving widow, or in the event of the boy's death as a minor, the whole estate was to be realised and, apart from certain annuities, was to be distributed between King Edward's Hospital Fund for London and other hospitals and charities. The unexpected has occurred, and within eight years of Mr. Charles Ansell's death the whole of these bequests—namely £263,500—falls into the lap of charity.

### MOUNT VERNON HOSPITAL BUILDINGS.

According to announcements the committee of Mount Vernon Hospital have completed the necessary arrangements for concentrating their ward cases at the Northwood institution. It is also stated that the hospital building and grounds at Hampstead have been acquired by the Medical Research Committee under the Insurance Act, and that they will be used for the purpose of laboratories and a central bureau. The changes necessitated at Northwood by the action of the governors have involved the creation of several special departments. Plans have also been prepared for the addition of 100 further beds, including a special wing for children, for which promises of support are understood to have been received. The work of the out-patient department in Fitzroy Square is unaffected and will continue.

### THE FUTURE OF BANSTEAD MENTAL HOSPITAL.

An important scheme of reconstruction of the Banstead Mental Hospital is foreshadowed by the Mental Hospitals Committee of the London County Council. The Committee point out that the main building of the hospital, which was erected before 1877 by the Justices for the county of Middlesex, was originally intended for the accommodation only of chronic patients, though it was soon found impossible to restrict it to this class of case. Its plan provides large wards which would be reasonably suitable for chronic cases, able-bodied and quiet, but which do not lend themselves at all well to the classification and supervision of the mixed population of an ordinary mental hospital. Nine of the blocks are very large, accommodating between them 1,535 patients. Each of these blocks contains three wards in three storeys, the ground floor serving as a day room and the two upper floors as dormitories. The day room often has to contain the whole population, sometimes as many as 180 patients, and the Committee are convinced that it is unreasonable to expect effective supervision or control of so large a number of patients in one room. They further feel that the congregation of so many mental patients in a ward seriously affects the comfort and contentment of all and the well-being of the hospital is prejudiced. The Committee are informed that the difficulty of classifying the various types of patients in these large blocks is very great, and that the medical officers find that patients after improving to convalescence in small wards reserved for first admissions, quickly show signs of relapse and have their complete recovery retarded after they have been removed, as is unfortunately necessary, to the large wards. Moreover, the steepness of the stairs leading to the upper dormitories makes it impossible for many old and feeble patients to be brought down into the airing courts. The Committee have felt strongly for some time that this state of affairs ought not to be allowed to continue. The outline of a scheme for structural alterations to remodel, at an approximate cost of £47,205, the nine large blocks to such an extent as to permit of better classification and treatment of their inmates has been approved by the Committee, and it is now proposed to spend a sum of £300 on the preparation of detailed plans and an accurate estimate of cost for further consideration. The Finance Committee approve the expenditure of £300 on the understanding that it is without prejudice to any action which they may take when the estimate for the works is before them.

### MUMPS IN THE CITY.

A number of cases of mumps having been reported among clerks in the City there has been, especially it seems on Christmas Eve, a rush of insurance against the risk of loss resulting from an attack. Those who had already had mumps were charged five shillings for a policy insuring £10 a week during each week of the coming three months for which they might be incapacitated, while the same benefits were obtainable at double the cost for those who had never previously contracted it. Insurance, beyond the limits of what may more properly be termed saving, has all the fascination of speculation for some people, and the attraction is a subtle one for many City persons, to whom it affords the interest of a hobby. Fancy policies, however, are not, we believe, as popular with the insurance companies as once they were, but the atmosphere in which they thrive is curiously different from that which affects most hospitals and institutions. Since writing the above the rates for mumps have increased, and we believe that some of the claims for benefits have already been presented.

### POSSIBILITY OF RABIES IN ENGLAND.

The outbreak of rabies in Savoy, with its sequel of manufactured alarm in this country, seems to show that on the whole the stringent regulations which the Board of Agriculture is empowered to make to restrict the importation of dogs into this country are as carefully enforced as ever. The order issued in 1901 provides that any dog brought into Great Britain from any other country except Ireland, the Channel Islands, and the Isle of Man shall not be landed unless a previous licence from the Board has been obtained. Six months' detention and isolation under the supervision of a veterinary surgeon in a place approved by the Board then follows at the owner's expense. All dogs are thus included, except genuine performing dogs and dogs intended for exportation within forty-eight hours of arrival in this country. In the face of these regulations and their enforcement one can hardly read without a smile the complaint of certain anonymous correspondents that they have known ladies every year to smuggle in undetected a pet dog in the amiable disguise of a baby or a handbag. Probably no laws are better enforced than those in question.

### THE SALE OF COCAINE SNUFF.

In a case recently heard in the Birmingham Police Court it was stated by one of the witnesses—an inspector under the Food and Drugs Acts—that cocaine snuff was commonly sold by tobacconists and licensed victuallers in West Bromwich. It is satisfactory to know that this practice is to be stopped by the prompt action of the Pharmaceutical Society. The sale of preparations of cocaine by any persons except registered chemists is an offence, and chemists who sell preparations of the kind are required to label the packages "Poison" and to observe the other conditions governing the sale of poisons. There appears to be no evidence that the practice which was said to be prevalent in West Bromwich is carried on in other parts of this country, and it is fortunate that the evil was discovered before it had had time to spread. In some other countries the cocaine habit has, in recent years, grown to an alarming extent, and it has been officially estimated that in the United States a hundred and fifty thousand ounces of cocaine are illegitimately used each year. For the present, at any rate, the machinery provided by the Pharmacy Acts seems all that is necessary to cope with the evil in this country.

### SANTA CLAUS IN A MONOPLANE.

At Addenbrooke's Hospital, Cambridge, on Christmas Day, about 3 p.m., a pierrot troupe commenced their entertainment in the wards, and were followed by a camel, lion, explorer, Suffragette, and a policeman, causing intense amusement. But a great surprise was ahead. In all the wards special teas were served, not only to the patients, but to the visitors present. As the tea in Victoria Ward was drawing to a close Father Christmas, Dr. Budd, house physician, arrived, to the great joy of all, by a Blériot monoplane. His arrival in this unexpected manner caused great excitement, and as soon as the crowd could be cleared from his plane by the police constable he proceeded to distribute the plentiful supply of gifts with which the giant Christmas-tree was loaded. Needless to say, the task of dismantling the tree kept Father Christmas busy for fully an hour and a quarter, and of necessity a certain amount of patience was demanded of all. And to make this demand less irksome to the children the camel was placed at their disposal, together with the explorer; and the children, making good use of the opportunity afforded them, tired the camel out. Thus ended one of the most original games of hospital Christmas this year of which we have yet heard. The Christmas festivities conclude with the nurses' fancy-dress dance and Advertisement Competition to-day, Saturday, when it is expected Miss Montgomery, the late matron, will be present.

### THE LATE SIR TREVOR LAWRENCE.

The death of Sir James John Trevor Lawrence at the age of eighty-two removes a well-known figure in the institutional world, and one who had practical experience both of the medical and administrative sides of hospital work. He succeeded not merely to his father's baronetcy, but to his father's profession—that of medicine, and to his father's hospital, St. Bartholomew's, of which in later life he became the well-known treasurer. After finishing his medical studies he entered the Indian Medical Service and served during the Mutiny till he returned to England in 1863. In 1875 he entered Parliament for Mid-Surrey, which he represented till he retired from the House in 1892. This led him to undertake the treasurership of St. Bartholomew's Hospital, where for twelve years of considerable difficulty he remained at his post. During this period the management of the hospital's estates underwent great changes, involving the abolition of a middleman to the financial advantage of the institution and to the greater satisfaction of the tenants. In addition the pathological block and the out-patients' department were rebuilt. The management, moreover, was adjusted so as to give a voice to the staff, in which important change Sir Trevor had a share. On the inauguration of King Edward's Hospital Fund for London he became a member of the Council on the recommendation of Lord Lister, and he also acted as vice-chairman of the Distribution Committee. He was created a K.C.V.O. in 1902, and was also a Knight of Grace of the Order of St. John of Jerusalem. It must be added that he took an active part in the rehabilitation of the Royal Horticultural Society, and was himself a well-known horticulturist and a distinguished grower of orchids.

### MODERN ABSURDITIES.

The British are becoming more and more a spoon-fed people. Paternal government is courted by many classes, and its ineptitudes are cruelly apparent. During the season before Christmas notices are issued by Government Departments—notably the Post Office—and by firms which are connected with distribution. How little effect such notices have in freeing postmen and parcel-deliverymen, and others connected with distribution and similar businesses, was brought home to us on Christmas Eve. An imposing package weighing some pounds was delivered in the late afternoon of December 24, and on examining it, it turned out to be a copy of a Directory for 1914. Christmas Day brought notices from the new Land Valuation Department of the Chancellor of the Exchequer, and some threatening letters to the clergy from the offices of the National Insurance Commission. Why the Chancellor of the Exchequer, or why anybody, for that matter, should wish such stuff to arrive at Christmas, or why its delivery should have been hurried to secure this end, is one of those mysteries which pass the understanding of knowledgeable experience? May we suggest that the staff of Carter-Paterson and the postmen have a very direct and unanswerable claim upon the Chancellor of the Exchequer and his officials on the Insurance Commission for the most handsome of Christmas boxes? Further, the public have good cause for grievance, seeing that the deliveries through the Post Office at this season grow worse and worse as the years pass.

### THE BRITISH HOSPITALS ASSOCIATION.

The fifth Annual Conference of this Association will be held at Newcastle-on-Tyne on Thursday and Friday, June 18 and 19, 1914. The Conference will be under the patronage of the Lord Mayor of Newcastle, who has extended his cordial support, and Mr. Roden Orde has kindly agreed to act as honorary local secretary. We have already expressed our satisfaction, with reasons, that the next Conference is to be held at Newcastle. The Chairman of the Council is Dr. D. J. Mackintosh, M.V.O., who has been medical superintendent of the Western Infirmary, Glasgow, for twenty-one years.

### THE GROWTH OF MEDICAL TRADE UNIONISM.

The status of the assistant medical officer has now emerged for discussion from the medical to the lay press, and it is noteworthy that the point seized on is that movement for combination among the assistant medical officers of public mental hospitals which was recently described in full in The Hospital. The truth is that from a variety of circumstances this new Association—the junior staffs of mental hospitals—is but the symptom of a general trend, and the year 1914 may well prove to mark the beginning of a general trade-union movement in modern medicine. Too long shy of this particular term, the profession has now been forced to face the fact that the despised term involves privileges not secured by any less frank title. The fact that the British Medical Association was the official organisation of the profession without being a registered trade union gave the National Medical Guild and more lately the National Medical Union their opportunity. We now see it announced that ships' surgeons are considering the possibility of combination on trade-union lines, and when these distinct branches of a profession begin to undertake combination for their respective concerns the ultimate recognition of trade-union principles in medicine appears inevitable. How far the new movement will succeed in mitigating the scarcity of medical men now complained of for resident posts of all classes, for the medical branches of the Services, for *locum tenentes*, and possibly for ships' surgeons, remains to be seen. That the welfare of the profession will be increased by closer co-ordination few will be found to-day to doubt. But co-ordination is not enough, is, indeed, worse than useless, as Dr. Nicholls shows in our correspondence columns this week, unless individuals intend to sacrifice themselves for the Associations that they have joined.

### SIR JOHN TUKE'S BEQUESTS.

An interesting and somewhat curious collection of bequests has been made by the late Sir John Batty Tuke, M.D., F.R.C.P., LL.D., F.R.S., D.Sc., the well-known chemist and former Unionist Member of Parliament for Edinburgh and St. Andrew's Universities. In addition to real estate, the testator left personal estate in the United Kingdom valued at £30,750, and as former President of the Royal College of Physicians, Edinburgh, bequeathed to it his bust by John Hutchison on condition that it be placed in the Great Hall of the College, and his etching of Darwin by Rayon. He also left to the Royal College of Surgeons the Dutch tiles which lined his study fire-place at Balgreen; while to the Æsculapian Club he bequeathed his antique silver-mounted Norwegian tankard and the silver cigar-box which Mrs. Charles Underhill had presented to him.

### INFIRMARIES AND THE INSURANCE ACT.

The National Insurance Act has been in force twelve months, and its effect upon Poor-Law infirmaries can now be realised. It was intended to keep these institutions outside the scope of the Act, but so many persons who are insured having found their way into the infirmaries, in any amendment of the Act their place in the health of the people will have to be considered. The general impression that the numbers in infirmaries would be reduced has not been realised—indeed, there is hardly any perceptible difference. The returns for four of the largest and poorest Poor Law Union areas south of the Thames bear this out. The inmates in the infirmaries were as follows at the close of each year:

| | 1912 | 1913 | Decrease | Increase |
|---|---|---|---|---|
| Camberwell ................ | 824 | 788 | 36 | — |
| Southwark ................ | 733 | 720 | 13 | — |
| Lambeth ................ | 578 | 598 | — | 20 |
| Wandsworth ................ | 1,217 | 1,233 | — | 16 |

In the four Unions there was a net decrease of thirteen patients in the infirmaries. On the other hand, there has been a remarkable decrease in the number of people receiving Poor-Law relief in its other varied forms. The figures show that 1,762 fewer people were assisted in 1913 than in 1912, and this is not due to old-age pensions, as the effect of the granting of the 5s. a week to the old folks has long since passed away. Why there should not have been a corresponding decrease in the inmates of infirmaries, due to better trade, as in the case of the outdoor poor, only those closely associated with Poor-Law work can answer. Their answer would be that the National Insurance Act has sent more people to the Poor-Law infirmaries, and that in the coming year their numbers would be bound to go up when the boom in trade has subsided.

### WATER ANALYSIS ON THE CHEAP.

It is encouraging to find that at any rate one public body exists which has quite given over belief in the "cash nexus." According to recent medical advertisements, the Taunton Rural District Council, being in want of a Medical Officer of Health, has decided to fix as that officer's yearly remuneration the sum of £110. Moreover, so far from the post being a sinecure, it makes mention that applicants should be capable of carrying out water analysis, this highly technical work being part of the duties appertaining to the post. The naïveté—we will put it down to that—of offering £2 a week for the care of the health of a largish district, with high chemical and bacteriological qualifications thrown in, is indeed truly rural. The population of the area is mentioned as being 17,000 odd. To complete the reduction to absurdity we might have been told the salary of the clerk to the Council who signs the notice. In all probability that gentleman would be very sorry to wield his fountain pen at the rate of remuneration laid down for the employment of chemical balance and Petri dishes and immersion lenses. The notice of this provincial conclave is a bull in every sense of the word—not least in having a particularly prominent bundle of hay on its horns.

### PATIENTS AND THEIR BEAUTY SLEEP.

In the very creditable, indeed quite Lipsian, article we published last week under "From the Patients' Point of View," there is a point mentioned which other on the whole grateful patients have grumbled a little about. That point, to put it tersely and colloquially, is the loss of their beauty sleep. Our British nursing system, which is strong enough to have been brought in as an argument against the introduction into this country of Continental methods of clinical research, seems to involve pretty constantly the waking of ward patients between the hours of 5 and 6 A.M. Our correspondent, with youthful iconoclasm, remarks that the morning toilette could have been performed quite as well an hour and a half later, adding with some cogency that a restless night spent in pain does not promote the keeping of early hours. We wonder if by now a generation of nursing authorities has arisen which can envisage this question with "a modern eye." The inmates of prisons, workhouses, perhaps convalescent homes, may surely be all the better for being gently constrained to shake off dull sloth, but sick men and women, accustomed all their life to take their sleep period from 11 P.M. to 8 A.M., cannot suddenly put their clock back some two and a half hours without inconvenience and suffering. Only the other day the secretary of a sanatorium remarked to us: "They say that up at the infirmary the patients never get any sleep at all." If it be only a case of rest and comfort *versus* appearances, then the case must go to the plaintiff, with costs on the higher scale. Will someone in the higher ranks of nursing favour us with her speech for the defence?

### FIFTY-TWO YEARS AT GUY'S HOSPITAL.

THE death occurred in December of Henry Williams, clerk to the Corporation of Guy's Hospital, within a few weeks of his seventieth birthday. This faithful and valued servant of the great hospital in the Borough had laboured fifty-two years within its walls, and he died in harness. The son of a Wiltshire clergyman, he was educated at Christ's Hospital, and on leaving school obtained employment with a firm of publishers. Thence he entered in 1861 the counting house of Guy's Hospital, and in 1882 he reached the headship of the department. In 1887, when Guy's was in sore straits for funds, it was Mr. Williams who bore the chief burden of an appeal to the public which brought in £100,000 in twelve months. Again, ten years later, he had to plead the same cause, once more with conspicuous energy and success. Beloved of all with whom he was brought into contact, Mr. Williams possessed a personality which endeared him to many generations of Guy's men. A certain eccentricity of attire was one of his characteristics, and he was one of those whom such a trait appears to render the more beloved on account of their foibles. A type of institutional official of the older school, he stood for all that is upright, dignified, picturesque. It will be long ere the memory of this genial and kindly man fades into oblivion at Guy's.

### ENDOWMENT OF BEDS BY INSTALMENTS.

PRACTICAL testimony of the interest displayed by work-people in the treatment of the sick has been shown in Leicester by a proposal from the work-people of Messrs. Waterhouse, Reynolds and Co. to endow a bed in the Leicester Royal Infirmary by spreading the amount of the endowment, £1,000, over a period of several years until the required sum is reached. As an earnest of their desire to complete the endowment as quickly as possible, an initial sum of £160 has been paid on account, and it is hoped that in seven years the endowment will be completed. This is the first proposal by a body of work-people to undertake the endowment of a bed in Leicester, and an example has been set which it is hoped may be followed by other employees. Endowment of beds by instalment is also an uncommon event, since collective bodies of people usually choose the form of "maintaining" a bed by a yearly subscription.

### LATEST REPORT OF ADDENBROOKE'S HOSPITAL.

THE latest report of Addenbrooke's Hospital, Cambridge, now a bulky and imposing volume of some 160 pages, has come to hand. Evidently Mr. Coles, known for his care in detail, has at length embodied the accumulated features suggested by experience into a more or less final form. The effect of the Insurance Act upon the Workers' Hospital Fund appears to have reduced the number of contributing centres from 112 to 94. Nevertheless, the Fund handed over £420 to the hospital, which was £57 less than in the previous year. It is worth noting that six months' experience of the Insurance Act decided the governors to proceed with the whole of the new out-patient department. The whole extension scheme, comprising the new children's wards, out-patient department, and new boiler-house, is expected to cost £25,000, of which £8,500 has yet to be raised. Unfortunately the hospital's income has shown a declining tendency, but now that it is so generally recognised how incomplete the Insurance Act would be without the hospitals, we cannot believe that this decrease will prove a permanent one.

### A HOSPITAL HISTORY CONTINUED.

SOME three years ago a history of St. George's Hospital upon a dignified scale was projected, and its publication in monthly parts was commenced. Subscriptions for the entire series of twelve fasciculi were invited and received; but after two parts had been published, the author, an old St. George's man, had a severe illness, and publication was inevitably deferred. From that time onwards, however, no further instalment of the history appeared, till Parts III. and IV., bound in one volume, reached us this week. The subscribers' grumblings, which have for a year or two been growing louder, should now have no cause for continuance. From an official statement in the *St. George's Hospital Gazette* we gathered that publication was about to be resumed, and that it was a committee which was to be responsible for this, not the original projector and author of the history. Hospital histories have our sympathy and appreciation, and indeed we retain a vivid remembrance of the promising start which was made in this particular case. To have allowed the history to remain in suspended animation would have been little to the credit of the patriotism of St. George's men; and readers of the first two parts will be glad to see that, committee or no committee, Dr. Peachey's name is still upon the cover.

### DR. MACKINTOSH'S "COMING OF AGE."

At a largely attended meeting on December 23 in connection with the Glasgow Western Infirmary the Chairman, Sir Matthew Arthur, congratulated Dr. Mackintosh, the medical superintendent, on the fact that in the twenty-one years that Dr. Mackintosh had held office he had rendered zealous and devoted service to the institution, which had been greatly appreciated by the managers, the staff, and the patients. Since 1892 the in-patients had more than doubled, the number of nurses had increased 100 per cent., and the household of the Western Infirmary now amounted to 879 souls. Sir Matthew Arthur said the board fully realised how much responsibility fell to the medical superintendent and the matron, Miss Gregory Smith. He rejoiced that they were able to bear this heavy burden and to flourish under it to the great advantage of the institution. The managers of the Western Infirmary have good reason to be satisfied with, and even proud of, their officers and staff.

### TRUTHFULNESS WITHOUT TACT.

Sir George Beatson made an excellent speech, in the course of which he told this story. Miss Nightingale taught that a nurse required not only knowledge, truthfulness, and all other virtues, but that she must have tact. One night a patient came into the hospital and was seen by the doctor, who left instructions with the nurse. The patient became delirious during the night and when the doctor arrived the next morning he asked when the man became so? The nurse said the man awoke about two o'clock in the morning and asked "When is that old fool coming in the morning?" (meaning the doctor). Then she added, "These were the only sensible words he has spoken since you paid your visit." No doubt a remarkable instance of strict truthfulness divorced from tact!

### THE DUST-FALL OF LONDON.

Every housewife complains of soot and dust, but she does not know how much she has to complain of! In London it is estimated that 51,000 tons fall yearly. This is no casual statement, but one verified by a carefully arranged gauge. Of course, this fall is not uniform. It is taken at four different stations: at Buckingham Gate, S.W., at Horseferry Road, S.W., at Old Street, E.C., and at Sutton, Surrey. The choice of these stations seems to the unenlightened mind a little arbitrary. But, taking the observations as we find them, we note that the total deposits in Sutton were 195 tons per square mile, while at Old Street they were 650 tons. In either case a sufficiently alarming amount! Worked out to an average over the 117 miles area of Greater London, we have an annual fall of 441 tons per square mile. And London is, as towns go, comparatively free from dust troubles. In Leeds, in the heart of the manufacturing district of the city, the annual fall of soot and dust is 539 tons, although in Roundhay, which is a residential part of the city, the dust-fall is only 25 tons per annum. Glasgow and the industrial towns in its neighbourhood have the worst record of all. In Glasgow the annual dust-fall is 1,330 tons per square mile, and in Coatbridge, which is the heart of the Scottish iron industry, it reaches the amazing total of 1,939 tons. One would wish for exact vital statistics to correlate with these of soot and dust, in order to know how far these impurities affect life. But at the best they have a most serious effect on vitality. It seems certain that when the significance of these figures is realised the efforts of public authority and private philanthropy will be directed actively to the control of the impure air, rather than to the treatment of diseases due to it.

### A GRAVE SCANDAL.

Messrs. T. Werner Laurie, Limited, have circulated widely an invitation to the public to send for, on approval, any book in their list entitled "Recent Notable Books." Amongst eleven books specially listed as notable appears "In a Cottage Hospital," a book which the whole hospital world has condemned as disgraceful and untrue. In support of its purchase is printed a notice from a nursing paper which declares, "It is not a book for the young person, but it is a book to be read." We regret exceedingly to notice a tendency in the journal in question towards prurient matter such as that this book contains. We are grateful to know that wholesome people on receiving this list of notable books, and noticing the book referred to, have thrown it in the fire with a resolve in self-defence to write "Caution" against books published by the firm in question.

### THIS WEEK'S DRUG MARKET.

Business has been at a standstill during the greater part of the week, and as stock-taking is now going on comparatively few orders will be placed until next week. The outlook, however, is quite hopeful. The value of opium is well maintained, and morphine and codeine are unchanged. An advance in the price of santonin may be announced at any moment notwithstanding the present extremely high quotation; not many years ago santonin could be purchased at about one-twentieth its present price, and there seems to be no end to the long series of advances; the present high price, which does not appear to have had the effect of checking the demand, is partly due to the fact that its manufacture is a monopoly and partly to scarcity of raw material. Atropine is dearer. Quinine is unchanged. Balsam of tolu tends higher in price. The market for cod-liver oil is very dull, and buyers are disposed to await reports of the earlier results of the new season's fishing in Norway before making their purchases.

